# Inhibition of miRNA-155 Alleviates High Glucose-Induced Podocyte Inflammation by Targeting SIRT1 in Diabetic Mice

**DOI:** 10.1155/2021/5597394

**Published:** 2021-03-08

**Authors:** Xiaolei Wang, Yanbin Gao, Wenming Yi, Yu Qiao, Hao Hu, Ying Wang, Yan Hu, Shuxin Wu, Hongfeng Sun, Taojing Zhang

**Affiliations:** ^1^Department of Endocrinology, Dongfang Hospital, Beijing University of Chinese Medicine, 6 Fangxingyuan, Fengtai District, Beijing, China; ^2^School of Traditional Chinese Medicine, Capital Medical University, 10 Youanmenwai, Xitoutiao, Fengtai District, Beijing, China; ^3^Beijing Key Lab of TCM Collateral Disease theory Research, 10 Youanmenwai, Xitoutiao, Fengtai District, Beijing, China

## Abstract

**Objective:**

Microinflammation plays a crucial role in podocyte dysfunction in diabetic nephropathy, but its regulatory mechanism is still unclear. This study is aimed at discussing the mechanisms underlying the effect of miRNA-155 on podocyte injury to determine its potential as a therapeutic target.

**Methods:**

Cultured immortalized mouse podocytes and diabetic KK-Ay mice models were treated with a miR-155 inhibitor. Western blotting, real-time PCR, ELISA, immunofluorescence, and Luciferase reporter assay were used to analyze markers of inflammation cytokines and podocyte injury.

**Results:**

miRNA-155 was found to be highly expressed in serum and kidney tissue of mice with diabetic nephropathy and in cultured podocytes, accompanied by elevated levels of inflammatory factors. Inhibition of miRNA-155 can reduce proteinuria and ACR levels, diminish the secretion of inflammatory molecules, improve kidney function, inhibit podocyte foot fusion, and reverse renal pathological changes in diabetic nephropathy mice. Overexpression of miRNA-155 *in vitro* can increase inflammatory molecule production in podocytes and aggravates podocyte injury, while miRNA-155 inhibition suppresses inflammatory molecule production in podocytes and reduces podocyte injury. A luciferase assay confirmed that miRNA-155 could selectively bind to 3′-UTR of SIRT1, resulting in decreased SIRT1 expression. In addition, SIRT1 siRNA could offset SIRT1 upregulation and enhance inflammatory factor secretion in podocytes, induced by the miRNA-155 inhibitor.

**Conclusions:**

These findings strongly support the hypothesis that miRNA-155 inhibits podocyte inflammation and reduces podocyte injury through SIRT1 silencing. miRNA-155 suppression therapy may be useful for the management of diabetic nephropathy.

## 1. Introduction

Podocyte dysfunction is prominent during proteinuria progression in several glomerular diseases [1]. Accumulating evidence suggests that microinflammation, a chronic low-grade inflammatory state that is involved in the pathological process of diabetic nephropathy (DN), leads to podocyte dysfunction [1–3]. When subjected to harmful stimuli, such as continuous chronic hyperglycemia, multiple metabolic pathways are disrupted. Gradually accumulating harmful products, such as advanced glycation end products (AGEs) and advanced oxidation protein products (AOPPs), can stimulate monocyte macrophages to secrete several inflammatory cytokines, including MCP-1, TNF-*α*, IL-1*β*, and IL-6. Inflammatory factor accumulation induces podocyte injury and promotes the further secretion of these inflammatory factors from podocytes, resulting in a vicious cycle that speeds-up disease progression [4]. Therefore, inhibition of inflammatory factor secretion can decrease podocyte injury and delay the progression of DN.

Noncoding RNAs such as microRNAs (miRs) are crucial posttranscriptional genetic modifiers. Recent literature has repeatedly emphasized that elevated miR-155 levels can be found in multiple kidney diseases [5–8]. A previous research study reported that miR-155 mediates pyroptosis of renal proximal tubular cells via targeting FoxO3a in renal ischemia-reperfusion injury [7]. Krebs et al. found that miR-155 stimulation in experimental crescentic glomerulonephritis resulted in accelerated kidney injury through T_H_17 immunity [8]. However, another study reported that miR-155 decreased the proliferation of mesangial cells in lupus nephritis, which indicates the renoprotective characteristics of miR-155 [9]. Nevertheless, the actual relationship between miR-155 and renal disease may be much more complex. Previous reports have linked miR-155 to high glucose-induced inflammation [10–12]. This series of experiments aimed at exploring the mechanism by which miR-155 regulates the response of podocytes to a high glucose environment and the regulatory mechanisms involved.

The pathological processes that drive podocyte dysfunction involve sirtuin 1 (SIRT1), a deacetylase that regulates cell inflammation, apoptosis, proliferation, autophagy, energy metabolism, and oxidative stress [13]. SIRT1 appears to exert a renoprotective effect in kidney diseases, including DN [14–16]. 3,5-Diiodothyronine can inhibit mesangial cell proliferation and decrease proteinuria by activating SIRT1 in rat mesangial cells [17]. Mice that possess SIRT1 knockout podocytes showed increased levels of urinary proteins [18]. Numerous studies have shown that 3′UTR region of SIRT1 can bind to miR-155 [19–21]. However, it is not known whether SIRT1 regulates inflammation in podocytes exposed to high glucose and whether this mechanism is associated with miR-155.

## 2. Materials and Methods

### 2.1. Reagents

Small interfering RNA (siRNA) against SIRT1, miR-155 mimics, and inhibitors, and negative controls were provided by Ribobio (China). Novus Biologicals (NBP1-77303, USA) provided the rabbit polyclonal anti-nephrin antibody. Rabbit polyclonal anti-SIRT1 antibody was purchased from Abcam (ab12193, USA). Mouse ELISA kits for IL-6 (CSB-E04639m), IL-1*β* (CSB-E08054m), TNF-*α* (CSB-E04741m), and MCP-1 (CSB-E07430m) were ordered from Cusabio (China).

### 2.2. Animals

KK-Ay mice (suitable for animal models of early to middle stage type 2 DN [22]) and C57BL/6J mice aged 8 weeks were obtained from the Chinese Academy of Medical Sciences (China). The mice were placed in a controlled 12 h light/dark cycle at constant humidity (70%) and room temperature (24°C). All animals were allowed free access to water. These mice were divided into three groups. The normal control group consisted of C57BL/6J mice (NC group, *n* = 8). KK-Ay mice were divided into a DN group (negative control, *n* = 8) and the DN treatment group (treated with miR-155 inhibitor, *n* = 8). The C57BL/6J mice were fed a standard diet, while the KK-Ay mice were fed a high fat diet. Both types of mice were maintained on this diet for 4 weeks, as previously documented [23]. Random blood glucose of ⩾16.7 mmol/L and a urinary albumin creatinine ratio (ACR) of ⩾300 *μ*g/mg were set as the diagnostic criteria for DN. The miR-155 inhibitor and its control were intravenously administered through the tail vein thrice a week for 3 weeks at a dose of 0.1 nmol/kg·d. After 3 weeks, 24-hour urine samples, serum, and renal tissues were harvested to be used in future experiments. The Animal Protection and Use Committee of Capital Medical University reviewed and approved all protocols.

### 2.3. Cell Culture

Conditionally immortalized mouse glomerular podocytes were procured from Chinese cell line infrastructure resources. RPMI 1640 medium (Genview, USA) enriched with 10% FBS (Sciencell, USA), 100 U/ml penicillin, 100 *μ*g/ml streptomycin and IFN-*γ* (10 to 20 U/ml) (Bioss, China) was used to culture the cells at 33°C in a 5% CO_2_ atmosphere. When the podocytes reached 80% confluency, the cells were passaged and stimulated to differentiate at 37°C in the absence of IFN-*γ* over a period of over seven days. Before proceeding to other experiments, the cells were maintained in a serum-free medium for 24 hours.

The cells were divided into the following groups: control cohort (11.1 mmol/L glucose); high glucose (HG) cohort (30 mmol/L glucose); miR-155 mimic or inhibitor cohort (50 nM miR-155 mimic or inhibitor); mimic or inhibitor normal control cohort (50 nM miR-155 mimic/inhibitor negative control); high glucose + miR-155 mimic/inhibitor or mimic/inhibitor control cohort; high glucose +miR-155 inhibitor + SIRT1 siRNA or control cohort (50 nM siSIRT1 or siRNA negative control). Cells in all groups were incubated with these reagents for 48 hours.

### 2.4. Immunofluorescence Staining

Renal tissues were fixed using 4% paraformaldehyde (Santa Cruz, USA) to produce paraffin (Sigma-Aldrich, USA)-embedded sections that were analyzed using immunofluorescence. The sections were first dewaxed and dehydrated to recover antigens. Endogenous peroxidase activity was halted using 3% hydrogen peroxide (Santa Cruz, USA) (diluted with PBS). Next, 5% goat serum (Thermo Scientific, USA) (diluted with PBS) was used to block the sections for 30 minutes. The samples were then incubated overnight with the nephrin antibody (1 : 100) (diluted with 5% goat serum) at 4°C, followed by incubation with the secondary antibody (Invitrogen, USA) (diluted with 1% BSA) for an hour at 37°C. Finally, the sections were stained with DAPI (Santa Cruz, USA). The stained cell images were produced using a fluorescence microscope.

### 2.5. Cell Transfection

Podocytes were seeded into a 6-well plate. A miR-155 mimic, inhibitor, or SIRT1 siRNA and their negative controls were used to transfect cells using 50 nM Lipofectamine 2000 (Invitrogen, USA). All procedures were conducted following the manufacturer's instructions.

### 2.6. Real Time PCR Analysis

Total RNA was extracted from podocytes, serum, and kidney tissues using TRIzol reagent (Invitrogen, USA). Previously established PCR protocols were used in this study [24]. Relative gene expression levels were determined using the comparative period threshold (CT) method (2^−ΔΔCT^) [25]. The relative expressions of nephrin and SIRT1 were compared with GAPDH, while that of miR-155 was compared to U6.

### 2.7. Western Blotting Analysis

Total protein extraction was performed on renal tissues and podocytes. An SDS-PAGE membrane was used to separate proteins before they were transferred onto a PVDF membrane (Merck Millipore, Germany). 5% nonfat dry milk (diluted with TBST) was used to block endogenous activity before the samples were subjected to overnight incubation with primary antibodies (diluted with 5% nonfat dry milk) at 4°C. The next morning, the samples were exposed to a horseradish peroxidase-conjugated secondary antibody (Cell Signaling Technology, USA) (diluted with 5% nonfat dry milk). The primary antibodies along with their dilutions were rabbit polyclonal antibody against nephrin (1 : 500) and SIRT1 (1 : 1000). Subsequently, signals were detected using enhanced chemiluminescence using a suitable reagent (Merck Millipore, Germany).

### 2.8. Luciferase Reporter Assay

PCR was performed to amplify both the mutant (MUT) and wild type (WT) 3′UTR of SIRT1 and then inserted into several cloning sites of the pmirGLO luciferase vector (Promega, USA) to construct SIRT1-WT and SIRT1-MUT vectors. The resultant vectors were cotransfected with either a miR-155 mimic or miR-155 mimic control in HEK293T cells using a Lipofectamine 2000 system (Invitrogen). At 48 h posttransfection, luciferase activity was assessed in all experimental cells.

### 2.9. Statistical Methods

All statistical analyses were conducted using SPSS software (IBM, USA). Data are presented as mean ± SD. The unmatched Student's *t* test was performed to assess variances between two groups. Differences among more than two groups were assessed using single-factor analysis of variance. A *P* value of less than 0.05 was interpreted to indicate statistical significance.

## 3. Results

### 3.1. The Effects of miR-155 on Inflammation, Renal Morphology, and Function in Diabetic KK-Ay Mice

**Figure 1:**
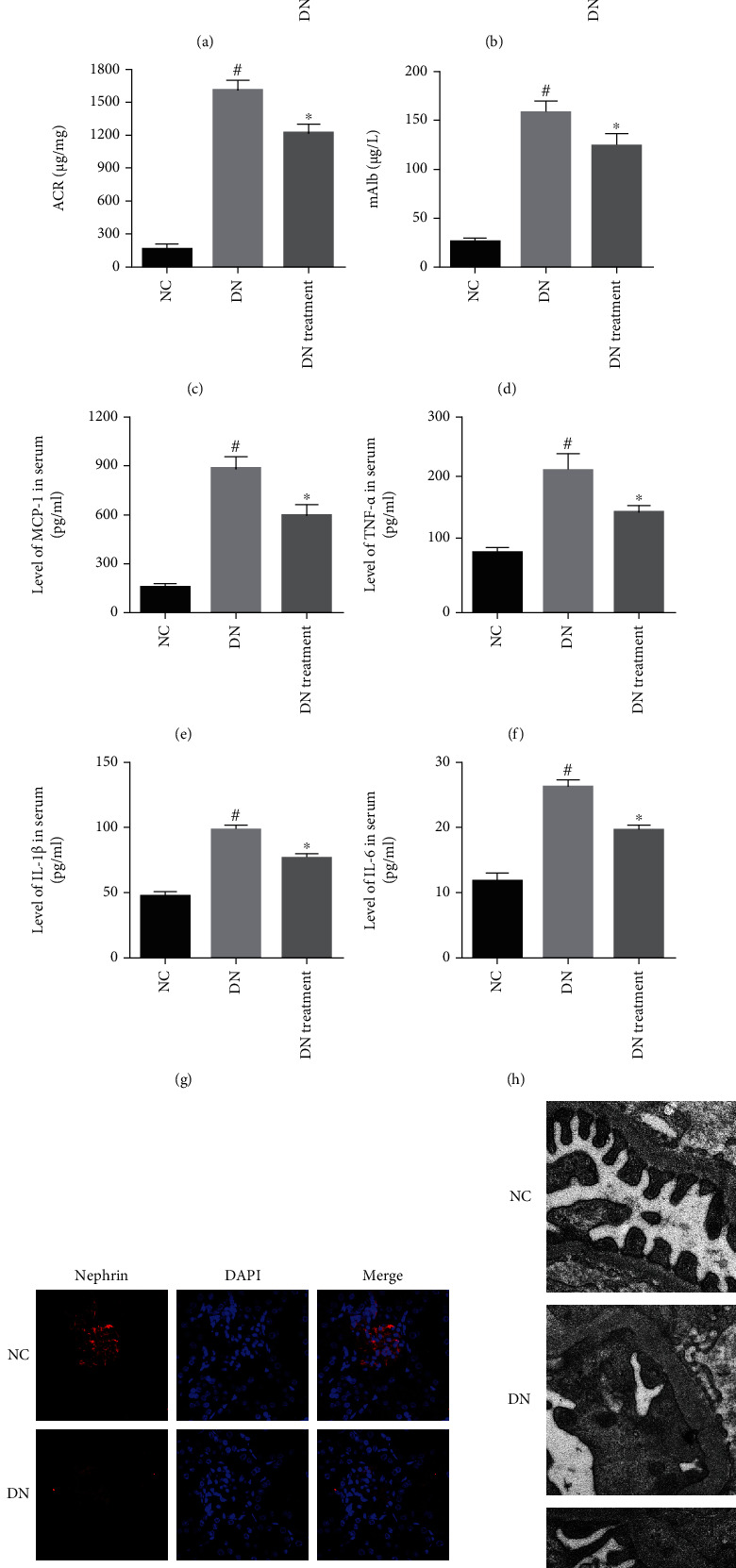
The impact of miR-155 on inflammation, renal morphology, and function in diabetic KK-Ay mice. (a, b) miR-155 expressions serum and kidney tissue as determined using real-time PCR. (c, d) ACR and mAlb levels were used to assess degree of renal function. (e–h) Inflammatory cytokine expressions (MCP-1, TNF-*α*, IL-1*β*, and IL-6) in serum was measured using ELISA. (i) The expression levels of nephrin were determined using immunofluorescence. (j) The morphology of the kidney was observed under TEM. ^#^Compared with NC group, *P* < 0.05; ^∗^Compared with DN control group, *P* < 0.05.

The effects of miR-155 expression in KK-Ay mice on ACR, microalbuminuria (mAlb), and renal morphology were investigated using serum tests for renal function and transmission electron microscopy (TEM). miR-155 expression in both the serum and renal specimens of DN mice were significantly elevated, while the converse was observed in DN mice treated with the miR-155 inhibitor (*P* < 0.05; Figures 1(a) and 1(b)). Microalbuminuria and ACR levels in the miR-155 inhibitor group of mice were comparatively lower (*P* < 0.05; Figures 1(c) and 1(d)) than the DN control cohort. Microinflammation is a common pathological process in DN [26]. Therefore, we quantified serum inflammatory factors (MCP-1, TNF-*α*, IL-1*β*, and IL-6). Mice with DN show significantly elevated serum inflammatory marker levels. Interestingly, exposure to a miR-155 inhibitor reversed this effect (*P* < 0.05; Figures 1(e)–1(h)). Moreover, we assessed the degree of podocyte injury using the expression level of nephrin, an established biomarker [27]. In contrast to untreated DN mice, miR-155 inhibitor intervention elevated nephrin levels (Figure 1(i)). Furthermore, TEM images demonstrate that miR-155 inhibitor intervention stimulated podocyte foot process fusion and improved podocyte structural disharmony, compared with untreated DN mice (Figure 1(j)). Therefore, miR-155 activity blockade appeared to relieve inflammation in the kidney, reduce podocyte injury, improve renal morphology and function of diabetic KK-Ay mice, and reduce renal fibrosis severity.

### 3.2. Effects of miR-155 on Inflammation Factors in Podocyte Injury Induced by High Glucose

**Figure 2:**
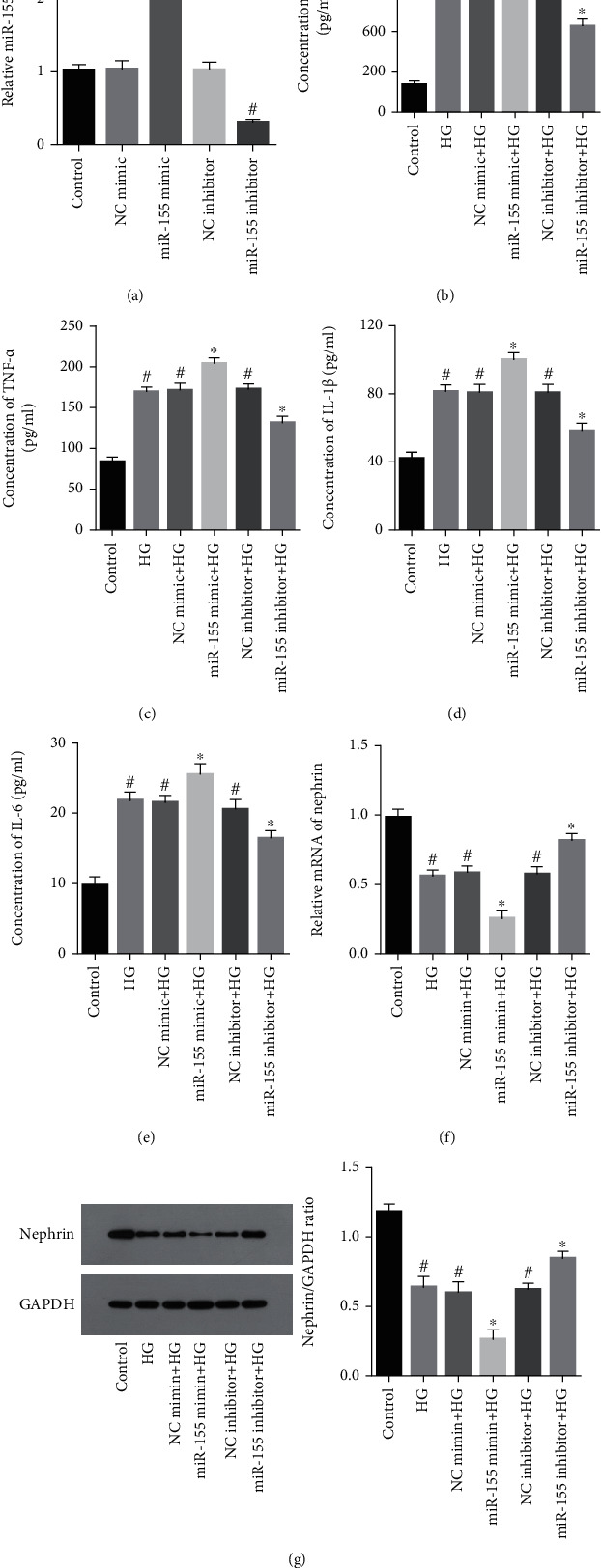
Effects of miR-155 on inflammation induced by high glucose-triggered podocyte damage. (a) The expression of miR-155 in podocytes transfected with the miR-155 mimic or inhibitor under high glucose condition determined using real-time PCR. (b–e) Inflammatory cytokines (MCP-1, TNF-*α*, IL-1*β*, and IL-6) secretion in podocytes transfected with the miR-155 mimic or inhibitor under high glucose conditions was assessed using ELISA. (f, g) Nephrin expression in podocytes transfected with either a miR-155 mimic or inhibitor was evaluated using real-time PCR and Western blotting analysis. ^#^Compared with control group, *P* < 0.05; ^∗^Compared with HG (high glucose) group, *P* < 0.05.

Real-time PCR allowed the relative miR-155 expression to be assessed to determine its association with glucose-induced podocyte injury and inflammation. Inflammatory cytokines (MCP-1, TNF-*α*, IL-1*β*, and IL-6) were noted to be raised in response to different conditions with miR-155 overexpression or silence for 48 h, as evidenced by ELISA. Simultaneously, podocyte injury marker (nephrin) level was measured using Western blotting and real-time PCR. Our data demonstrated that miR-155 and inflammatory cytokines were overexpressed in podocytes exposed to high glucose conditions (*P* < 0.05; Figure 2(a) and *P* < 0.05; Figures 2(b)–2(e), respectively). Overexpressed miR-155 further increased the secretion of these inflammatory molecules but decreased the level of nephrin in a high glucose environment, while miR-155 silencing reversed these effects (*P* < 0.05; Figures 2(b)–2(g)). In conclusion, miR-155 can aggravate inflammatory factor release in high glucose-induced podocyte injury. Suppression of miR-155 expression appears to reverse inflammation-mediated podocyte injury induced by high glucose conditions.

### 3.3. SIRT1 Is a Direct Target of miR-155 in Podocytes

**Figure 3:**
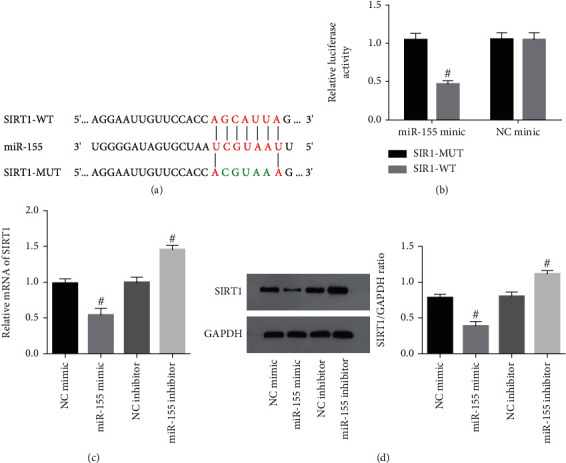
miR-155 directly targeted SIRT1 in podocytes. (a) miR-155 binding site specific for SIRT1. (b) Luciferase activities of HEK-293T cells that overexpressed miR-155 along with either WT-SIRT1 or MUT-SIRT1. ^#^Compared with NC mimic group, *P* < 0.05. (c, d) SIRT1 expressions in podocyte with miR-155 overexpression or suppression was assessed using real-time PCR and Western blotting analysis. ^#^Compared with NC mimic or inhibitor group, *P* < 0.05.

To understand the miR-155-mediated mechanism by which podocyte inflammation is regulated, we carried out a bioinformatics analysis. We found SIRT1 to be a potential target gene involved in the inflammatory response, as it contains a predicted miR-155 binding site (Figure 3(a)). HEK-293T cells transfected with the miR-155 mimic demonstrated decreased wild-type SIRT1 luciferase activity. On the other hand, transfection with a mutant SIRT1 did not yield this effect (*P* < 0.05; Figure 3(b)). Furthermore, overexpression of miR-155 decreased SIRT1 expression in podocytes, while miR-155 inhibition enhanced SIRT1 podocyte expression (*P* < 0.05; Figures 3(c) and 3(d)). The results highlight the direct association between SIRT1 and miR-155, with miR-155 regulating the expression of SIRT1 in podocytes.

### 3.4. The Effects of miR-155 Silencing on Podocyte Inflammation Is Reversed by the Downregulation of SIRT1

**Figure 4:**
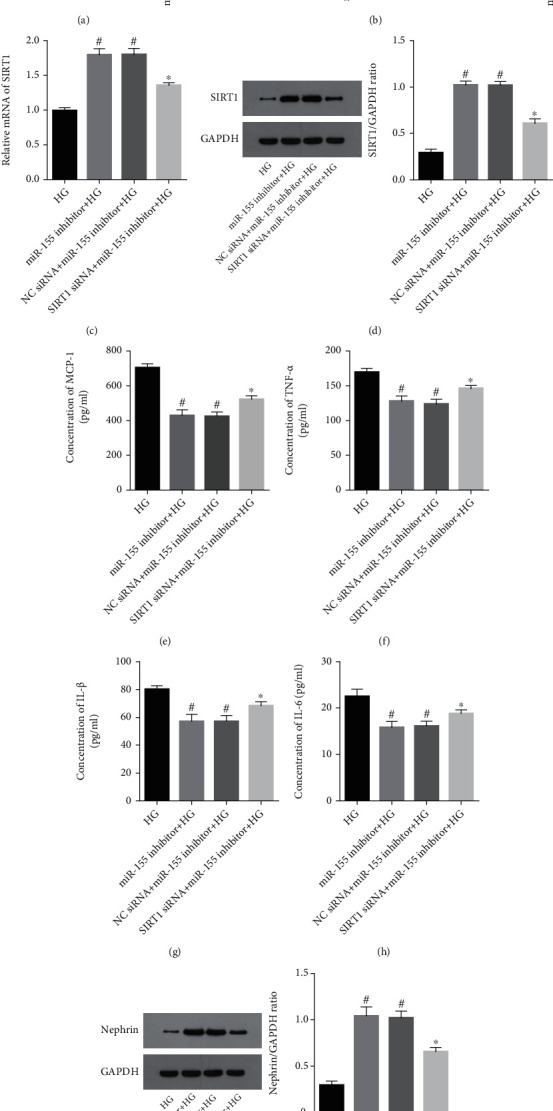
The effects of miR-155 silencing on podocyte inflammation was reversed by SIRT1 downregulation. (a, b) SIRT1 expression in podocytes transfected with the miR-155 mimic or inhibitor under high glucose conditions was evaluated using real-time PCR and Western blotting analysis. ^#^Compared with control group, *P* < 0.05; ^∗^Compared with HG (high glucose) group, *P* < 0.05. (c, d) The expression of SIRT1 in podocytes transfected with the miR-155 inhibitor and SIRT1 siRNA under high glucose conditions was assessed using real-time PCR and Western blotting analysis. ^#^Compared with HG (high glucose) group, *P* < 0.05; ^∗^Compared with miR-155 inhibitor + HG group, *P* < 0.05, the same below. (e–h) Inflammatory cytokine expressions (MCP-1, TNF-*α*, IL-1*β*, and IL-6) in podocytes transfected with the miR-155 inhibitor and SIRT1 siRNA under high glucose conditions was determined using ELISA. (i) Nephrin expression in podocytes cotransfected with the miR-155 inhibitor and SIRT1 siRNA under high glucose conditions was quantified using Western blotting analysis.

To further verify whether SIRT1 was crucial for miR-155-mediated podocyte inflammation, both the miR-155 inhibitor and SIRT1 siRNA were cotransfected into podocytes exposed to high glucose conditions. Our data shows that SIRT1 protein expression in podocytes cultured in a high glucose environment decreased, with the reverse was confirmed in the presence of the miR-155 inhibitor (*P* < 0.05; Figures 4(a) and 4(b)). Podocyte expression of SIRT1 in the miR-155 inhibitor and SIRT1 siRNA cotransfected podocytes were lower compared with cells only transfected with the miR-155 inhibitor (*P* < 0.05; Figures 4(c) and 4(d)). SIRT1 downregulation counteracted the increase in SIRT1 induced by the miR-155 inhibitor. Moreover, SIRT1 downregulation markedly suppressed the secretion of inflammatory factors (MCP-1, TNF-*α*, IL-1*β*, and IL-6) in podocytes exposed to the miR-155 inhibitor (*P* < 0.05; Figures 4(e)–4(h)). Meanwhile, SIRT1 downregulation inhibited nephrin upregulation triggered by the miR-155 inhibitor (*P* < 0.05; Figure 4(i)). Our results suggest that SIRT1 inhibition reversed the effects of miR-155 silencing on podocyte inflammatory factors in a high glucose environment.

## 4. Discussion

DN appears to be strictly associated with certain miRNAs during its pathophysiological process [28–30]. DN patients may benefit from therapy targeting DN-associated miRNAs. This study revealed that miR-155 was significantly elevated in serum and kidney specimens of DN mice and podocytes exposed to a high glucose environment. Microinflammation mediates an important function in DN podocyte injury [31, 32]. This provided the basis of determining the relationship between miR-155 expression and the podocyte inflammatory response. miR-155 inhibition can suppress inflammatory factor secretions in the serum of DN mice. In podocytes cultured in a high glucose environment, inhibition of miR-155 can inhibit podocyte inflammatory factor production, and alleviate podocyte injury, while miR-155 upregulation exerted an enhanced inflammatory reaction and enhanced the degree of podocyte injury.

Several studies have reported that miR-155 plays a role in diabetes and its complications through the regulation of targeted genes. Wei et al. reported that miR-155 promotes insulin resistance in obese mice via targeting PPAR*γ* [33], while Lin et al. reported that miR-155 inhibits insulin resistance in diabetic mice via several negative regulators (C/EBP*β*, HDAC4, and SOCS1) [34]. Ji et al. reported that the downregulation of miR-155 inhibited apoptosis and alleviated inflammation in rat Schwann cells via targeting Nrf2 [35]. Shen et al. reported that miR-155 aggravates endothelial progenitor cell dysfunction via targeting PTCH1 [36]. Bioinformatics analysis methods were used to determine the target genes responsible for regulating the mechanism by which miR-155 stimulated the podocyte-induced release of inflammatory factors. SIRT1 is a candidate that was strongly associated with inflammation. It has been reported that miR-155 regulates biological processes by targeting SIRT1. In mice with myocardial ischemia/reperfusion injury, upregulation of miR-155 increases infarction size and myocardial cell apoptosis via targeting SIRT1 [19]. In human nasal epithelial cells, silencing of miR-155 inhibits epithelial-to-mesenchymal transition via targeting SIRT1 [37]. In mice with obstructive nephropathy, miR-155 aggravates renal interstitial fibrosis via targeting SIRT1 regulation [38]. In our research, SIRT1 was confirmed to represent a target gene of miR-155 through luciferase experiments. SIRT1 is an important metabolic regulator that participates in the inflammatory response by regulating acetylation levels of histone and transcription factors, such as AP1 and NF-*κ*B [39]. SIRT1 is considered as a potential target for the treatment of diabetes and its complications [40]. In this study, we confirmed that SIRT1 expression is negatively regulated by miR-155. Moreover, SIRT1 silencing eliminated miR-155 inhibition after inflammatory factor release in podocytes in a high glucose environment.

To conclude, these findings demonstrated that miR-155 expression was elevated in podocytes cultured under high glucose conditions. miR-155 appeared to promote the release of inflammatory factors in podocytes, aggravating podocyte injury by targeting SIRT1. At the same time, inhibition of miR-155 can decrease ACR and proteinuria in DN mice, improve renal function, inhibit podocyte foot process fusion, and improve renal pathological features. The miR-155/SIRT1 axis found through this study lays the foundation for further experiments on the molecular mechanism of podocyte injury and highlights that miR-155 may be a novel target for DN treatment.

## Data Availability

The data used to support the findings of this study are available from the corresponding author upon request.
